# Quality of recovery after laparoscopic cholecystectomy: a randomized trial of pneumoperitoneum pressure and neuromuscular blockade depth

**DOI:** 10.1016/j.bjane.2025.844667

**Published:** 2025-07-29

**Authors:** José Fernando Amaral Meletti, Marina Gasparotto Fernandes, Eduardo Toshiyuki Moro, Evaldo Marchi

**Affiliations:** a Faculdade de Medicina de Jundiaí, Departamento de Cirurgia, Jundiaí, SP, Brazil; b Faculdade de Medicina de Jundiaí, Pós-Graduação em Ciências da Saúde, Jundiaí, SP, Brazil; c Pontifícia Universidade Católica de São Paulo (PUC-SP), Faculdade de Medicina, Departamento de Cirurgia, São Paulo, SP, Brazil

**Keywords:** Cholecystectomy, Laparoscopy, Neuromuscular blockade, Postoperative pain

## Abstract

**Introduction:**

Laparoscopic Cholecystectomy (LC) is a commonly performed surgical procedure. The pneumoperitoneum and the depth of Neuromuscular Blockade (NMB) may impact the occurrence of postoperative pain and the quality of recovery.

**Methods:**

A randomized, double-blind, and prospective clinical trial with 124 patients undergoing LC, divided into 4 groups: SP/MB (Standard Pneumoperitoneum pressure and Moderate NMB); LP/MB (Low Pneumoperitoneum pressure and Moderate NMB); SP/DB (Standard Pneumoperitoneum pressure and Deep NMB); and LP/DB (Low Pneumoperitoneum pressure and Deep NMB). Recovery quality was assessed using the Quality of Recovery Questionnaire (QoR-40), and postoperative pain was evaluated using a Verbal Numerical Rating Scale (VNRS).

**Results:**

No difference was observed between groups regarding the total QoR-40 score 24 hours after surgery (p = 0.903). Despite better surgical conditions (scored from 0 to 5) in the LP/DB group (4.7 ± 0.52) and lower in the LP/MB group (4.1 ± 0.95), the LP/DB group showed a longer stay in the Post-Anesthesia Care Unit (PACU), a higher need for rescue treatment for nausea and vomiting in the ward (p = 0.044), and greater resting pain at 24 hours (p = 0.027).

**Conclusion:**

The use of different pneumoperitoneum pressures under moderate or deep neuromuscular blockade in patients undergoing Laparoscopic Cholecystectomy (LC) did not alter patients’ perception of postoperative recovery quality. The combination of standard pneumoperitoneum pressure with deep neuromuscular blockade was associated with a better perception of surgical field quality as evaluated by the surgeon.

## Introduction

Laparoscopic Cholecystectomy (LC) is one of the most commonly performed surgical procedures worldwide.^1^ Although it is a short-duration surgery, many patients still experience unexpectedly prolonged hospital stays or readmissions due to difficult-to-control postoperative pain.^2^

Several studies have sought to propose possible alternatives for preventing postoperative pain. One of the most researched interventions is the use of low-pressure pneumoperitoneum compared to standard pressure.^3^ However, the reduction of effective working space provided by lower intra-abdominal pressure can increase technical difficulty, the incidence of procedure-related injuries, and the duration of the surgery.^4^

Another variable to consider is the depth of Neuromuscular Blockade (NMB). Deep NMB can improve surgical conditions by facilitating visualization and manipulation of intra-abdominal structures. The limitation for the use of deep NMB in the past was the lack of agents capable of reversing the blockade quickly and predictably. However, this limitation was eliminated with the advent of sugammadex. Many studies have evaluated the benefits of deep NMB on patient's pain intensity and postoperative recovery in patients undergoing laparoscopic surgeries.^5^ However, it is unclear whether these positive effects result from lower pneumoperitoneum pressure, the depth of NMB, or both, further studies are needed to clarify aspects related to postoperative recovery.

Traditionally, perioperative studies have focused on post-operative outcomes such as time to wakening, hospital length of stay, nausea, vomiting and pain control. Measurements that assess quality of life from patient´s perspective are increasingly recognized as important in clinical studies that aim to investigate the effect of anesthesia and surgery on patient recovery and satisfaction. One such tool is the Quality of Recovery Questionnaire (QoR-40) which is validated 40-item scoring system developed to asses many aspects of post-surgical recovery.^6^

The hypothesis of the present study was that low-pressure pneumoperitoneum combined with deep neuromuscular blockade, compared to standard-pressure pneumoperitoneum and moderate neuromuscular blockade, would be able to improve the quality of postoperative recovery as assessed by the Quality of Recovery-40 (QoR-40) questionnaire after laparoscopic cholecystectomy. Accordingly, this study aims to compare the quality of recovery in patients undergoing elective laparoscopic cholecystectomy under low-pressure pneumoperitoneum (10 mmHg) and standard pressure (14 mmHg) associated with either deep or moderate neuromuscular blockade. The following secondary outcomes were also considered: surgical conditions, occurrence of postoperative pain, nausea and vomiting, and analgesic consumption.

## Methods

This double-blind, randomized, and prospective clinical trial was approved by the Research Ethics Committee (CAAE 42586621.9.3001.5447) and registered with the Brazilian Registry of Clinical Trials (ReBEC) under U1111-1265-2384. The informed consent was obtained voluntarily from each patient. A total of 132 participants aged between 18 and 65 years, classified as physical status according to the American Society of Anesthesiologists (ASA) I and II, were included and underwent general anesthesia for elective LC at the Regional Hospital of Jundiaí-SP. Data were collected during the period from May to October 2022.

The exclusion criteria before randomization were: (I) Patient refusal; (II) Altered consciousness level or inability to communicate; (III) Contraindications to the use of any agent described in the protocol; (IV) Alcohol or drug abuse; (V) Body Mass Index (BMI) ≥ 35, which could impact the safety of surgeries under the low-pressure pneumoperitoneum protocol; (VI) Chronic pain or opioid use; (VII) Neuromuscular disease; (VIII) Complicated cholelithiasis. Exclusion criteria after randomization included: (I) Protocol violation; (II) Conversion to open surgery; and (III) Patient refusal in the postoperative period.

The participants were randomly allocated into four distinct groups using a random number generator (www.random.org): Group SP/MB (standard Pneumoperitoneum pressure and Moderate NMB); Group LP/MB (Low Pneumoperitoneum pressure and Moderate NMB); Group SP/DB (Standard Pneumoperitoneum pressure and Deep NMB); and Group LP/DB (Low Pneumoperitoeum pressure and Deep NMB). The randomization sequence was stored by a non-research participant and revealed only when all data were forwarded for statistical analysis. For each patient, two opaque envelopes were prepared (one containing the pneumoperitoneum pressure and the other describing the degree of NMB), sealed and sequentially numbered. The envelopes were opened at the time of surgery by an independent nurse who was not involved in patient care or data collection. Neither the patient, surgeon, nor anesthesiologist involved in data collection knew which group each patient belonged to. The degree of NMB was only known by the anesthesiologist responsible for anesthesia.

The study participants did not receive pre-anesthetic medication, as it could negatively influence completion of the Quality of Recovery-40 questionnaire prior to surgery. Age, sex, ASA physical status, and BMI were recorded. Anesthesia induction was performed with sufentanil (0.5 µg.kg^-1^), propofol (2.0 mg.kg^-1^), and rocuronium (0.45 mg.kg^-1^), 1.5 × ED95. Patients in the deep NMB groups (SP/DB and LP/DB) received an additional dose of rocuronium (0.45 mg.kg^-1^) two minutes after intubation (total of 3 × ED95). NMB was monitored using acceleromyography (TOF Watch SX®; Schering-Plough). The Train-Of-Four (TOF) was evaluated at 15-second intervals by analyzing the response to stimulation of the ulnar nerve, aiming to maintain 1‒3 responses in the moderate NMB groups and no response (Post-Tetanic Count [PTC] of 1‒3) in the deep NMB groups. Additional doses of 5 to 10 mg of rocuronium were used to maintain the TOF and PTC according to the depth of NMB previously determined. Anesthesia maintenance was performed with sevoflurane (1.5%–3%). After incision at each trocar insertion site, infiltration with 0.75% ropivacaine (total volume 20 mL) was performed by the surgical team. The abdomen was insufflated with carbon dioxide to maintain intra-abdominal pressure at 10 mmHg (LP group) or 14 mmHg (SP group), according to the group determined by randomization. The pneumoperitoneum pressure levels were determined based on previous studies^3^^,^^4^ and the participating surgeons agreed with the protocol. The display showing the insufflation pressure was obscured so that only the room nursing staff had access to this information. All patients received dexamethasone (10 mg), ketoprofen (100 mg), dipyrone (2000 mg) and ondansetron (4 mg). After the procedure, atropine (0.01 mg.kg^-1^) and neostigmine (0.04 mg.kg^-1^) were administered for patients in the moderate NMB group and sugammadex 4 mg.kg^-1^ for those in the deep NMB group. After awakening, extubation was performed. The time from the end of surgery to awakening was recorded, as well as the surgical time. Surgical conditions were evaluated by surgeons according to an ordinal scale: 1 (extremely poor conditions), 2 (poor conditions), 3 (acceptable conditions), 4 (good conditions), and 5 (excellent conditions).

Pain intensity was assessed at rest and recorded every 15 minutes during the stay in the Post-Anesthesia Care Unit (PACU) using a Verbal Numeric Scale (VNRS) from 0 to 10. Morphine 1‒2 mg intravenously was administered every 5 minutes to achieve a score below 4 (1 mg for pain < 7 and 2 mg for pain ≥ 7). After discharge from the recovery room, all patients received ketoprofen 100 mg every 12 hours and paracetamol 500 mg orally every 6 hours. Pain intensity was evaluated upon arrival at the ward, 4 h, 8 h, 12 h, and 24 hours after surgery using the VNRS. In cases of insufficient analgesia, tramadol (100 mg) was offered. Postoperative Nausea and Vomiting (PONV) were treated with dimenhydrinate (30 mg), which was considered as rescue medication. The use of rescue medications and the occurrence of postoperative nausea and vomiting were recorded. All patients remained in the hospital for at least 24 hours.

The primary outcome was the quality of recovery on the first day after surgery, assessed using the Quality of Recovery Questionnaire (QoR-40) in its version validated for Brazilian Portuguese. Interviews were conducted twice for each patient: before surgery and in the ward 24 hours after surgery, carried out by a member of the research team trained and knowledgeable in administering the questionnaire. It was not necessary to consider the Minimum Clinically Important Difference (MCID) in our study because, for the QoR-40 questionnaire, this value is 6.3.^7^ Our results did not reach this threshold.

Sample size calculation was based on a similar randomized clinical trial that assessed postoperative recovery quality using the QoR-40 questionnaire in patients undergoing abdominal hysterectomy with different anesthetic techniques,^8^ considering an alpha error of 0.05 and a power of 90% to detect a 10-point difference in QoR-40, requiring the inclusion of 31 patients per group. A 10-point difference represents a 15% improvement in recovery quality based on previously reported values in QoR-40. Considering potential losses, the final sample size included 132 patients.

Categorical variables were expressed as absolute values for frequency comparison (percentages) and analyzed using the Chi-Square test. Quantitative variables, whose results were not normally distributed according to the Kolmogorov-Smirnov test, were compared using the Kruskal-Wallis test to simultaneously compare all four groups. When a difference was found between groups, the Mann-Whitney test was used for pairwise comparisons to determine where the difference occurred. A post hoc analysis for multiple comparisons (Bonferroni correction) was performed to more accurately determine the differences between groups. Pre- and postoperative moments were compared separately for all scores (paired data) using the Wilcoxon test. A significance level of 5% and a 95% Confidence Interval were considered for all tests. For this statistical analysis, the software SPSS version 20 and Minitab 16 were used.

## Results

**Figure 1:**
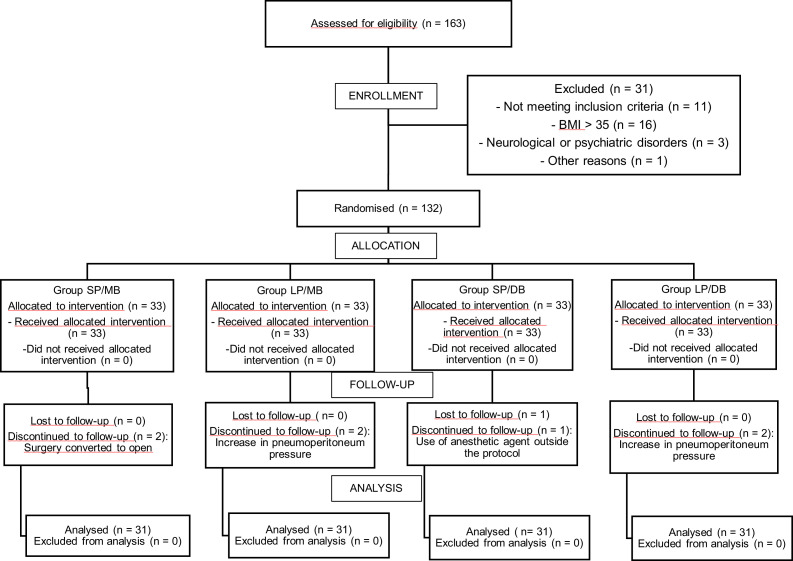
CONSORT flow diagram of patient selection and allocation.

A total of 163 patients were considered eligible to participate in the study. Of these, 31 were excluded before randomization. One hundred thirty-two patients were randomly allocated into four groups and received the intervention. Eight patients were excluded from the study after randomization, resulting in 124 participants for analysis (Fig. 1).

The groups were considered homogeneous with respect to demographic and perioperative characteristics, except for the surgical field conditions as evaluated by the surgeons (p = 0.039). The mean score was significantly higher in the SP/DB group (4.71 ± 0.52) and lowest in the LP/MB group (4.12 ± 0.95), with values expressed as median and interquartile range in Table 1.

**Table 1:** Demographic and perioperative characteristics.

	Group SP/MB (n = 31)	Group LP/MB (n = 31)	Group SP/DB (n = 31)	Group LP/DB (n = 31)	p-value
**Age (years)**	47 (34‒53)	52 (42‒57)	47 (38‒55)	47 (38‒57)	0.563
**ASA**					0.965
I	12 (38.7%)	12 (38.7%)	11 (35.5%)	13 (41.9%)	
II	19 (61.3%)	19 (61.3%)	20 (64.5%)	18 (58.1%)	
**BMI (kg.m^-2^)**	30.90 (27.00‒32.07)	28.20 (24.62‒30.06)	28.00 (26.15‒30.38)	29.70 (26.09‒31.30)	0.217
**Female gender**	25 (80.6%)	25 (80.6%)	28 (90.3%)	26 (83.9%)	0.698
**PONV Risk**	2 (1‒2)	2 (1‒2)	2 (2‒2)	2 (2‒2)	0.280
**Wake up time (min)**	10 (7‒14)	12 (10‒15)	10 (10‒15)	10 (10‒15)	0.167
**Surgery time (min)**	55 (45‒64)	55 (50‒73)	60 (50‒65)	60 (50‒71)	0.377
**Surgical conditions**	5.0 (4.0‒5.0)	4.0 (3.5‒5.0)	5.0 (4.5‒5.0)	5.0 (4.0‒5.0)	0.039
**Adverse events**	3 (9.7%)	1 (3.2%)	1 (3.2%)	3 (9.7%)	0.659

Results expressed in Median (interquartile range) or frequency of occurrence (%).

PONV, Postoperative Nausea and Vomiting; Risk (risk factors 0 to 4), Simplified Apfel Score.

The data related to the scores obtained according to the QoR-40 are described in Table 2. Both the total score and those obtained for the different domains were similar across the groups.

**Table 2:** Dimensions of the Quality of Recovery-40 (QoR-40) Questionnaire by Study Groups before surgery and 24 hours after surgery.

	Group SP/MB (n = 31)	Group LP/MB (n = 31)	Group SP/DB (n = 31)	Group LP/DB (n = 31)	p‒value
**Before surgery**					
Comfort	56 (55‒58)	58 (56‒60)	57 (55‒59)	58 (58‒60)	0.257
Emotional state	42 (38‒43)	43 (40‒44)	43(42‒44)	43 (41‒44)	0.131
Physical independence	20 (19‒20)	20 (20‒20)	20 (20‒20)	20 (20‒20)	0.004
Psychological support	40 (39‒40)	40 (40‒40)	40 (39‒40)	40 (40‒40)	0.160
Pain	34 (32‒35)	34 (33‒35)	35 (34‒35)	35 (35‒35)	0.519
TOTAL	189 (186‒194)	195 (189‒197)	194 (189‒198)	195 (191‒198)	0.050
**24 hours after surgery**					
Comfort	58 (56‒59)	58 (55‒59)	58 (55‒59)	58 (57‒59)	0.928
Emotional state	43 (41‒45)	44 (43‒45)	44(43‒45)	44 (42‒45)	0.297
Physical independence	20 (19‒20)	20 (18‒20)	20 (19‒20)	20 (18‒20)	0.725
Psychological support	40 (40‒40)	40 (40‒40)	40 (40‒40)	40 (40‒40)	0.669
Pain	33 (32‒34)	33 (32‒34)	33 (32‒34)	33 (32‒34)	0.903
TOTAL	194 (188‒197)	193 (188‒197)	194 (190‒197)	193 (190‒196)	0.938

Results expressed in median (interquartile range).

The parameters obtained in the PACU and during the stay in the ward are described in Table 3. The patients' time in the PACU was significantly longer for those in the SP/DB group (p = 0.010) compared to the other groups. Pain intensity at rest 24 hours after surgery was significantly higher in the SP/DB group (p = 0.027).

**Table 3:** Parameters in the Post-Anesthesia Care Unit (PACU) and in the ward.

	Group SP/MB (n = 31)	Group LP/MB (n = 31)	Group SP/DB (n = 31)	Group LP/DB (n = 31)	p-value
**Pain PACU**					
Arrival	0 (0‒0)	0 (0‒0)	0 (0‒0)	0 (0‒0)	0.472
15 min.	0 (0‒0)	0 (0‒0)	0 (0‒1)	0 (0‒0)	0.501
30 min.	0 (0‒0)	0 (0‒0)	0 (0‒2)	0 (0‒0)	0.052
45 min.	0 (0‒0)	0 (0‒0)	0 (0‒2)	0 (0‒0)	0.054
**Morphine (mg) – PACU**	0 (0‒0)	0 (0‒0)	0 (0‒0)	0 (0‒0)	0.219
**PONV in PACU**	2 (6.5%)	4 (12.9%)	2 (6.5%)	1 (3.2%)	0.676
**PACU time (min.)**	30 (30‒45)	32 (30‒45)	45 (41‒60)	43 (30‒45)	0.010
**Pain Ward**					
Arrival	0 (0‒4.5)	0 (0‒0)	0 (0‒1)	0 (0‒0)	0.216
4 hours	0 (0‒1)	0 (0‒0)	0 (0‒3)	0 (0‒1.5)	0.548
8 hours	0 (0‒0)	0 (0‒0)	0 (0‒0)	0 (0‒0)	0.779
12 hours	0 (0‒0)	0 (0‒0)	0 (0‒0)	0 (0‒0)	0.576
24 hours	0 (0‒0)	0 (0‒0)	0 (0‒0)	0 (0‒0)	0.027
**Pain medication in Ward**	5 (16.1%)	4 (12.9%)	6 (19.4%)	6 (19.4%)	0.919
**PONV in Ward**	2 (6.5%)	2 (6.5%)	8 (25.8%)	6 (19.4%)	0.092
**PONV medications in Ward**	1 (3.2%)	2 (6.5%)	8 (25.8%)	6 (19.4%)	0.044

Results expressed in Median (interquartile range) or number (percentage).

PONV, Postoperative Nausea and Vomiting.

Regarding the use of antiemetics in the ward, it was observed that the patients in the deep NMB groups had a greater need for rescue medication (p = 0.04).

## Discussion

Patients subjected to lower pneumoperitoneum pressures appear to experience less postoperative pain intensity after laparoscopic surgeries. However, the decrease in the effective working space provided by lower intra-abdominal pressure may increase technical difficulty, the incidence of procedure-related injuries, and surgery duration.^9^ The appropriate relaxation of the diaphragm and abdominal muscles with deep NMB could mitigate this issue and even reduce postoperative pain intensity.^10^ To our knowledge, no author has yet investigated which variable (pneumoperitoneum pressure and/or NMB) could improve the recovery quality of patients undergoing Laparoscopic Cholecystectomy (LC) without compromising the quality of the surgical field visualization.

In the present study, patients subjected to 10 or 14 mmHg pneumoperitoneum pressure with or without deep NMB were compared. According to the scores obtained through the QoR-40 application, neither low pneumoperitoneum pressure, deep NMB, nor the combination of both variables were able to improve recovery quality (total score or each domain) within 24 hours after LC. Özdemir-van Brunschot et al.^11^ evaluated recovery quality in patients undergoing laparoscopic nephrectomy with low pneumoperitoneum pressure associated with deep NMB and concluded that this technique was unable to alter the QoR-40 score.

Regarding the use of deep Neuromuscular Blockade (NMB) to improve postoperative recovery in laparoscopic surgeries, Torensma et al.^12^ evaluated patients undergoing laparoscopic bariatric surgery and observed that deep NMB was able to reduce postoperative pain intensity. Yang et al.^13^ applied the Quality of Recovery-15 questionnaire, a simplified version of the QoR-40, in patients undergoing laparoscopic bariatric surgery and found higher QoR-15 scores and lower pain intensity scores. On the other hand, two other studies failed to demonstrate such benefit.^14^^,^^15^ In our study, deep neuromuscular blockade did not result in improved postoperative recovery quality as assessed by the QoR-40 questionnaire and did not reduce pain following laparoscopic cholecystectomy. According to a recent consensus published by the European Society of Anaesthesiology, there is insufficient evidence to support the use of deep NMB for the purpose of reducing postoperative pain.^16^

Pneumoperitoneum results in an acutely elevated intra-abdominal pressure. Patients with morbid obesity have chronically elevated abdominal pressures. During laparoscopy in morbidly obese patients, the pneumoperitoneum pressure should not be lower than 15 mmHg in order to provide adequate visualization and exposure of the operative field.^17^ Therefore, we excluded patients with a BMI ≥ 35. A systematic review found that low-pressure pneumoperitoneum in laparoscopic surgeries was associated with lower pain scores, assessed using the Numeric Rating Scale, compared to standard pressure during the first two postoperative days.^18^ However, in the present study, low-pressure pneumoperitoneum did not improve the evaluated parameters of postoperative recovery quality compared to standard pressure. A Cochrane systematic review revealed a high risk of bias and low or very low quality of evidence in 20 out of 21 studies analyzed, providing no justification to support the use of low-pressure pneumoperitoneum in patients undergoing elective laparoscopic cholecystectomy.^19^

Pneumoperitoneum results in a state of acutely elevated intraabdominal pressure. Similar to nonobese subjects, the intraabdominal pressure during laparoscopy of the morbidly obese is set at 15 mmHg to provide adequate visualization and exposure of the operative field. The normal intraabdominal pressure of nonobese individuals is 5 mmHg or less. In contrast, morbidly obese patients have a chronically elevated intraabdominal pressure at 9 to 10 mmHg. This section discusses the physiologic effects in increased intraabdominal pressure during pneumoperitoneum on femoral venous flow and renal, hepatic and respiratory function.

In a meta-analysis, the authors concluded that reduced pneumoperitoneum pressure combined with deep NMB was not significantly more effective than moderate NMB to optimize the surgical space conditions and postoperative pain. This review did not evaluate recovery quality after laparoscopic surgery.^20^ Another recent study comparing patients undergoing LC under different pneumoperitoneum pressures with deep NMB found no difference between groups regarding recovery quality.^21^ Although it did not improve recovery quality, deep NMB in patients with Standard Pneumoperitoneum pressure (SP/DB group) provided better surgical field visualization according to the surgeons' opinion. As expected, the combination of low pneumoperitoneum pressure with moderate NMB resulted in the opposite effect, i.e., worse surgical field quality. These findings are in line with previous studies that recommend deep neuromuscular blockade to optimize intraoperative conditions when visualization is suboptimal.^5^^,^^12^, ^13^, ^14^^,^^16^ Martini et al.^11^ and Rosenberg et al.^20^ also demonstrated that deep NMB improves the surgeon's perception of the surgical field quality compared to moderate NMB.

Other variables were assessed. Pain intensity in the first 24 hours postoperatively and the need for antiemetic rescue were greater among patients subjected to standard pneumoperitoneum pressure and deep NMB (SP/DB group). Although these findings are statistically significant, we do not consider them to have substantial clinical relevance. In the individualized evaluation of patients at the final postoperative assessment, two patients reported a pain intensity score of 2, which is considered mild, and this accounted for the observed statistical difference. Considering the assessment of the Minimum Clinically Important Difference (MCID) in postoperative pain studies, we know that it varies considerably, being influenced by patients’ baseline pain, definitions of clinical pain improvement, and study design. In this context, the definition of an MCID value is highly individualized.^22^ In the present study, the MCID was based on the authors’ consensual judgment. Regarding the increased use of rescue medication for PONV, we believe this finding lacks clinical significance, especially since the statistical result was close to the conventional threshold (p = 0.044). We were unable to find a plausible explanation for this statistical finding. The results observed in these two variables were not sufficient to reduce the overall quality of recovery in these patients.

This study has some limitations. First, the recovery quality was limited to the first 24 hours postoperatively, and it would be interesting to know the impact of the different interventions on the following days. Second, cases where the protocol was violated were excluded, and the distribution of these patients according to the “intention-to-treat” principle was not applied. Third, the sample size was calculated to evaluate the primary outcome (recovery quality) but not for other outcomes, such as pain intensity or NVPO incidence. Fourth, this study was conducted at a single university hospital, and a multicenter evaluation is needed for these data to be safely extrapolated to the general population. Finally, the assessment of NMB depth was not blinded. However, as the primary outcome was evaluated by an independent researcher and on the following day, we believe this did not interfere with the results.

## Conclusion

The use of pneumoperitoneum pressures of 10 or 14 mmHg under moderate or deep neuromuscular blockade did not significantly affect the quality of recovery in patients undergoing laparoscopic cholecystectomy, as assessed by the QoR-40 questionnaire. However, deep neuromuscular blockade under standard pneumoperitoneum pressure improved surgical field conditions as evaluated by the surgeons, although it was associated with increased postoperative pain and a greater need for antiemetics. Future studies are necessary to validate these findings and expand the available evidence.

## Funding

I declare that the research was funded with personal resources and that the authors have no relevant conflicts of interest related to this research.

## Trial registration

Brazilian Registry of Clinical Trials (ReBEC) U1111-1265-2384. Date: 26/09/2024

https://ensaiosclinicos.gov.br/observador/submissao/sumario/11131.

Protocol and statistical analysis plan: josemeletti@g.fmj.br

Data sharing: josemeetti@g.fmj.br

## Authors' contributions

José Fernando Amaral Meletti: Study design, data collection, data interpretation, manuscript drafting, and final approval of the version to be published.

Marina Gasparotto Fernandes: Study design, data collection, data interpretation, manuscript review, and final approval of the version to be published.

Eduardo Toshiyuki Moro: Study conception and design, data interpretation, manuscript review, and final approval of the version to be published.

Evaldo Marchi: Data interpretation, manuscript review, and final approval of the version to be published.

## Conflicts of interest

The authors declare no conflicts of interest.

## References

[1] Hayden P., Cowman S. (2011). Anaesthesia for laparoscopic surgery. Contin Educ Anaesth Crit Care Pain.

[2] Rosero E.B., Joshi GP. (2017). Hospital readmission after ambulatory laparoscopic cholecystectomy: incidence and predictors. J Surg Res.

[3] Vijayaraghavan N., Sistla S.C., Kundra P. (2014). Comparison of standard-pressure and low-pressure pneumoperitoneum in laparoscopic cholecystectomy: a double blinded randomized controlled study. Surg Laparosc Endosc Percutan Tech.

[4] Gurusamy K.S., Vaughan J., Davidson BR. (2014). Low pressure versus standard pressure pneumoperitoneum in laparoscopic cholecystectomy. Cochrane Database Syst Rev.

[5] Park S.K., Son Y.G., Yoo S., Lim T., Kim W.H., Kim JT. (2018). Deep vs. moderate neuromuscular blockade during laparoscopic surgery: A systematic review and meta-analysis. Eur J Anaesthesiol.

[6] Myles P.S., Weitkamp B., Jones K., Melick J., Hensen S. (2000). Validity and reliability of a postoperative quality of recovery score: the QoR-40. Br J Anaesth.

[7] Myles P.S., Myles D.B., Galagher W., Chew C., MacDonald N., Dennis A. (2016). Minimal Clinically Important Difference for Three Quality of Recovery Scales. Anesthesiology.

[8] Catro-Alves L.J.S., De Azevedo V.L.F., De Freitas Braga T.F., Goncalves A.C., De Oliveira GS. (2011). The effect of neuraxial versus general anesthesia techniques on postoperative quality of recovery and analgesia after abdominal hysterectomy: a prospective, randomized, controlled trial. Anesth Analg.

[9] Martini C.H., Boon M., Bevers R.F., Aarts L.P., Dahan A. (2014). Evaluation of surgical conditions during laparoscopic surgery in patients with moderate vs deep neuromuscular block. Br J Anaesth.

[10] Staehr-Rye A.K., Rasmussen L.S., Rosenberg J. (2014). Surgical space conditions during low-pressure laparoscopic cholecystectomy with deep versus moderate neuromuscular blockade: a randomized clinical study. Anesth Analg.

[11] Özdemir-van Brunschot D.M.D., Scheffer G.J., van der Jagt M. (2017). Quality of Recovery After Low-Pressure Laparoscopic Donor Nephrectomy Facilitated by Deep Neuromuscular Blockade: A Randomized Controlled Study. World J Surg.

[12] Torensma B., Martini C.H., Boon M. (2016). Deep Neuromuscular Block Improves Surgical Conditions during Bariatric Surgery and Reduces Postoperative Pain: A Randomized Double Blind Controlled Trial. PloS One.

[13] Yang W.L., Wen Y.L., Xu W.M., Xu C.L., Yin W.Q., Lin JY. (2024). Effect of deep neuromuscular block on the quality of early recovery after sleeve gastrectomy in obese patients: a randomized controlled trial. BMC Anesthesiol.

[14] Rosenberg J., Herring W.J., Blobner M. (2017). Deep Neuromuscular Blockade Improves Laparoscopic Surgical Conditions: A Randomized, Controlled Study. Adv Ther.

[15] Choi B.M., Ki S.H., Lee Y.H. (2019). Effects of depth of neuromuscular block on postoperative pain during laparoscopic gastrectomy: A randomized controlled trial. Eur J Anaesthesiol.

[16] Fuchs-Buder T., Romero C.S., Lewald H. (2023). Peri-operative management of neuromuscular blockade: A guideline from the European Society of Anaesthesiology and Intensive Care. Eur J Anaesthesiol.

[17] Nguyen N.T., Wolfe BM. (2005). The physiologic effects of pneumoperitoneum in the morbidly obese. Ann Surg.

[18] Ortenzi M., Montori G., Sartori A. (2022). Low-pressure versus standard-pressure pneumoperitoneum in laparoscopic cholecystectomy: a systematic review and meta-analysis of randomized controlled trials. Surg Endosc.

[19] Gurusamy K.S., Vaughan J., Davidson BR. (2014). Low pressure versus standard pressure pneumoperitoneum in laparoscopic cholecystectomy. Cochrane Database Syst Rev.

[20] Wei Y., Li J., Sun F., Zhang D., Li M., Zuo Y. (2020). Low intra-abdominal pressure and deep neuromuscular blockade laparoscopic surgery and surgical space conditions: A meta-analysis. Medicine (Baltimore).

[21] Moro E.T., Pinto P.C.C., Neto AJMM (2021). Quality of recovery in patients under low- or standard-pressure pneumoperitoneum. A randomized controlled trial. Acta Anaesthesiol Scand.

[22] Olsen M.F., Bjerre E., Hansen M.D. (2017). Pain relief that matters to patients: systematic review of empirical studies assessing the minimum clinically important difference in acute pain. BMC Med.

